# Caring for Adolescents and Young Adults (AYA) with Cancer: A Scoping Review into Caregiver Burdens and Needs

**DOI:** 10.3390/cancers15123263

**Published:** 2023-06-20

**Authors:** Milou J. P. Reuvers, Asiye Gedik, Kirsty M. Way, Sanne M. Elbersen-van de Stadt, Winette T. A. van der Graaf, Olga Husson

**Affiliations:** 1 Division of Psychosocial Research and Epidemiology, Netherlands Cancer Institute, 1006 BE Amsterdam, The Netherlands; 2 Department of Medical Oncology, Erasmus MC Cancer Institute, Erasmus University Medical Center, 3015 GD Rotterdam, The Netherlandsw.vd.graaf@nki.nl (W.T.A.v.d.G.); o.husson@nki.nl (O.H.); 3 School of Heath Sciences, University of Southampton, Southampton SO17 1BJ, UK; 4 Independent Researcher, 1006 BE Amsterdam, The Netherlands; 5 Department of Medical Oncology, Netherlands Cancer Institute—Antoni van Leeuwenhoek, 1006 BE Amsterdam, The Netherlands; 6 Department of Surgical Oncology, Erasmus MC Cancer Institute, Erasmus University Medical Center, 3015 GD Rotterdam, The Netherlands

**Keywords:** informal caregivers, AYA cancer patients, caregiver burden

## Abstract

**Simple Summary:**

Informal caregivers are an underrepresented group in the AYA literature, as attention is often paid to the patients’ wishes and needs. Caring for young cancer patients brings age-specific challenges, which are related to the life-phase of the patients. This causes these caregivers to differ from those caring for children and older patients. This review aimed to provide an overview of the impact of being a caregiver for an AYA cancer patient on different domains of their lives, as well as to report the unmet needs they identify. By being more attentive to the caregivers and their needs, and intervening accordingly (e.g., via social support or psychological interventions), caregivers’ quality of life can increase, which allows for better caregiving capabilities. This not only has a positive impact on the patient, but might also lead to a decreased caregiver burden.

**Abstract:**

AYAs with cancer (aged 15 to 39 at primary diagnosis) form a specific group within oncology, and there is limited information on the impact on their informal caregivers. This scoping review aimed to gain insight into the burden on caregivers of AYAs with cancer and identify the unmet needs they might have. Eligible articles focused on impacts in one of the domains of caregiver burden (physical, psychological, social, on schedule, financial) or unmet needs. In all domains of caregiver burden, impact was reported by caregivers. Caregiving leads to physical problems (such as sleep problems) and psychological symptoms (e.g., depression, anxiety, and negative emotions). Loneliness is reported, and little peer-support. Many different tasks and roles must be undertaken, which is perceived as challenging. In addition, there is a financial impact and there are unmet needs to be met. Several domains of the lives of caregivers of AYA cancer patients are negatively affected by the disease. Some of these are age-specific, and tailored to a particular group of caregivers (parents, partners, or friends). AYA cancer patients represent a wide age range, resulting in the engagement of many different caregivers. Future research will need to take this into account in order to adequately provide support.

## 1. Introduction

Adolescence and young adulthood are challenging stages in one’s life, characterized by many unique physical, emotional, cognitive, and social changes [1]. During this period, a transformation occurs in which adolescents and young adults (AYAs) move towards autonomy, become independent from their parents, form an identity, explore intimacy, finish education, and start their working careers and their own family. A cancer diagnosis can disrupt these normative processes [2]. AYA cancer patients are those diagnosed with cancer between the ages of 15 and 39 years old, and they form a distinct population within oncology [3]. Consequently, AYA cancer patients have age-specific needs, such as age-adapted information, and a focus on sexuality and fertility, and also on education or career. In the past decade, age-specific care programs have been developed for AYAs with cancer [4].

Handling a cancer diagnosis at such a young age might be extra challenging, as cognitive capabilities and emotional maturation are not fully developed, especially among younger AYAs. As a result, it can be difficult to integrate living with a disease like cancer into normal life [5]. Studies show that support from significant others, such as family or friends, positively contributes to help AYAs to live with their disease. Interactions with others can reduce the stress of living with cancer and increase patients’ adaptation skills. Nevertheless, distress and concerns about their disease (e.g., fear of progression, physical (late) effects, questions on prognosis) can occur and might have a negative impact on the quality of life of AYAs with cancer [6]. Patients, therefore, frequently rely on their informal caregivers for practical, emotional, or medical support [7]. 

Consequently, a cancer diagnosis at a young age does not only affect the patient, but also their family and social network. These informal caregivers, encompassing romantic partners, parents, siblings, friends, or colleagues, are the initial source of help for patients and offer them support [8]. With a few exceptions, AYAs are mostly treated in an outpatient setting. Therefore, more care takes place at home, leading to an increased burden on the informal caregivers. The number of caregiving responsibilities might vary, but these are often handled in addition to the responsibilities that caregivers already have, including their job, homework, and care for other children or family members [8]. Aside from practical help, informal caregivers are often part of the communication between the patient and the healthcare professional, and assist in making decisions when it comes to treatment options. If there are physical symptoms due to cancer or its treatment, a caregiver may be involved in managing these symptoms by providing medical care or seeking information on possible support for any discomfort. Furthermore, given the emotional burden of a cancer diagnosis, caregivers often provide moral support to patients [9]. For partners, challenges arise when the cancer diagnosis impacts their shared future together, for example due to infertility of the patient. This may lead to anxiety, depressive symptoms, grief, or a reduced quality of life. In addition, the potential fertility concerns could have practical implications for fertility preservation procedures that may affect the patient and their partner [10].

The responsibilities of caring for an AYA cancer patient can lead to caregiver burden. Caregiver burden is defined as “the extent to which caregivers perceive that their physical health, psychological health, schedule, social life, and financial status have suffered due to providing care for a cancer patient” [11]. The literature shows that caregivers generally report a higher prevalence of anxiety than the cancer patients, mainly because they worry about losing someone important to them because of the disease. A study on caregiver needs reported that many caregivers identify a strong need for emotional help and assistance in maintaining attention to their own needs [12]. A study by Hu et al. (2018) found that taking care of a younger cancer patient causes an increased risk for caregiver burden as, aside from the impact of a cancer diagnosis, other issues occur, e.g., the confrontation with death or infertility at a young age. Young cancer patients and their caregivers tend to be more overwhelmed by cancer in comparison to those who are older, because of the age-related challenges, and thus tend to be prone to increased stress related to their disease [13]. Furthermore, since cancer is diagnosed at a young age and curative treatment is occurring more frequently, survivorship care may take many years. This long-term care could increase the burden on caregivers taking care of AYA cancer patients, since this may affect them over a longer period of time, and this may have an impact on their own regular daily activities [14]. Informal caregivers are an underrepresented group in the AYA literature, as attention is often paid to the patients’ wishes and needs. Junkins and colleagues stress the need for specific insights into the impact for caregivers of these patients, as this is not yet available [14]. Additionally, the type of caregivers who support AYA cancer patients are sometimes different from those caring for older patients (e.g., parents). Therefore, it is necessary to identify the impact on a caregiver of an AYA cancer patient. 

This review aims to provide an overview of (1) the impact of caring for an AYA cancer patient and (2) specific informal caregivers’ needs. By gaining insight into the impact on caregivers, adequate help can be offered and potentially added to the current AYA healthcare programs. This might improve the quality of life for caregivers and patients, and allow caregivers to appropriately support their AYA. 

## 2. Materials and Methods

This review was reported according to the PRISMA 2020 guidelines (Supplementary Data S1) [15]. 

### 2.1. Search Strategy

A search of the literature was performed in the databases Medline, via Ovid, Embase.com, PsycInfo, via Ovid, and CINAHL, via Ebsco. Results were restricted to studies published from January 2000 onwards. Conference abstracts were excluded based on the publication type in Embase.com. This search was performed on the 28 October 2022. The search strategy used both text word and thesaurus terms where appropriate. Search strategy terms combined young adults AND cancer AND informal caregivers AND burden; synonyms were also included. The full search strategy can be found in Supplementary Data S2. The searches were checked by a second information specialist. The results were deduplicated using EndNote, first based on the PMIDs and then by following steps A through D of the Bramer dedup method [16]. Forward and backward snowballing was conducted, to include other relevant publications from the reference lists. The search yielded 3172 results. 

### 2.2. Selection Criteria

**Figure 1:**
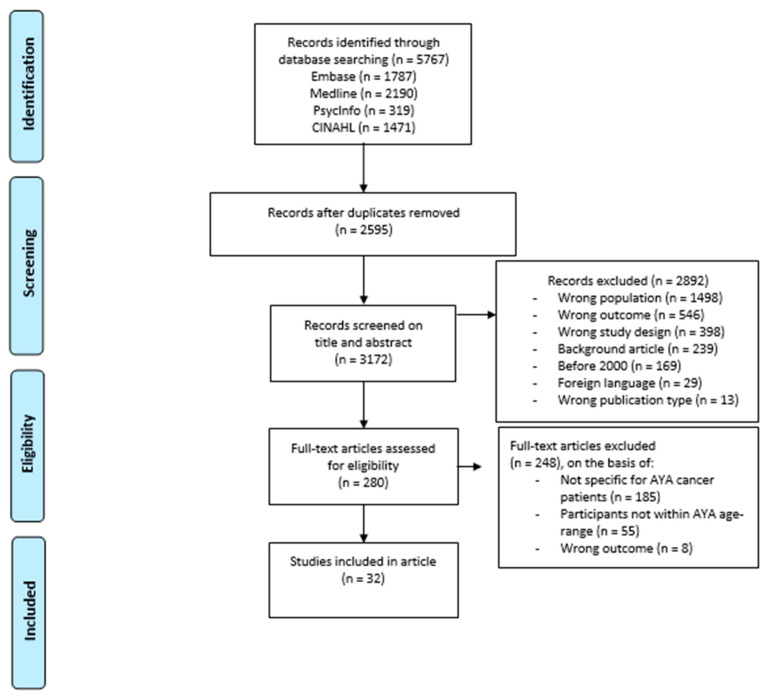
Flow chart of article inclusion.

Studies that met the following criteria were included in the analysis: (1) if the objective of the study was to determine the impact of being an informal caregiver of an AYA cancer patient (aged 15 to 39) or a description of those caregivers’ needs, (2) if the publication was an original article, (3) if the article was published in English, (4) if the article was published in a peer-reviewed journal, (5) if the article mentioned an age-range in the abstract, and (6) if the full text was available. The inclusion and exclusion criteria were applied to the 3172 initial results. The titles and abstracts of all articles were reviewed by two independent reviewers (MR and AG) using Rayyan (www.rayyan.ai, accessed on 18 June 2023). This yielded 280 eligible articles. Full texts were then screened, resulting in 32 full texts which met the inclusion criteria and were therefore included in the review. Figure 1 shows the flow chart describing the selection procedure.

### 2.3. Data Extraction 

Study design, characteristics of the respondent, outcome measures, and the findings regarding the impact on caregivers were extracted from the included articles. The findings were divided into the domains of caregiver burden: biological, psychological, social, schedule, financial, or unmet needs. The distributions of the data collected were consensual between two researchers (MR and OH).

## 3. Results

In total, 32 studies were included, all published between 2002 and 2022. The primary outcome of all studies was the impact of caregiving for an AYA cancer patient, or caregivers’ unmet needs. The results were reported by the definition of caregiver burden and the life domains it had an impact on: biological, psychological, social, schedule, financial, and unmet needs. The main findings are summarized in Table 1, Table 2, Table 3, Table 4, Table 5 and Table 6. For qualitative studies, the topics were identified from the Section 3 of the original article. Caregivers are reported in this study as they are mentioned in the original article. If this is not specified, the specific type of caregiver is unknown.

### 3.1. Biological Impact

Eleven studies mentioned the biological impact (i.e., physical health) of caring for a young patient with cancer (Table 1). A mixed-methods study by Stevens et al. (2018) reported that 78% of 28 networkers (those whom patients identified as important during their illness trajectory) identified an impact on their physical wellbeing during the process of caregiving. Networkers mostly included parents, friends, and partners [17]. In several studies, participants indicated that taking care of young cancer patients leads to fatigue and feeling exhausted [18,19,20,21]. In one study, being fatigued caused a partner to be apathetic, as they had little energy left to express emotions [20]. Feeling tired also had an impact on concentration throughout the day, as experienced by parents [21]. Contributing to the care of a young cancer patient was not the sole cause of fatigue. Two studies cited difficulties associated with sleep [22,23]. The sleep of siblings was in some cases disturbed after the cancer diagnosis, given their concern about what the consequences of this disease might be on the patient [23]. Family caregivers also reported sleeping problems due to overthinking. These difficulties primarily came from being busy with what had to be arranged for the patient (e.g., where the patient had to go or what medication had to be given). In addition, caregivers often had little time to rest between responsibilities, and therefore woke up earlier to complete all of their tasks [22]. 

In addition to fatigue and sleeping problems, family caregivers (partners, siblings, and mothers) reported additional physical complaints that they related to caring for a patient. A qualitative study identified back pain, abdominal pain, headaches, anorexia, and insomnia as recurring symptoms [18]. For some caregivers, physical complaints and symptoms that were present prior to the AYA’s diagnosis were often given less attention or completely ignored. This often led to the worsening of the caregiver’s symptoms [24]. 

The disease also had an impact on sexual health. In a cross-sectional study of 289 partners of young breast cancer patients, 20% reported they were no longer sexually active with their partner [25]. Another study on partners of female cancer patients also reported problems with sexual health, causing partners to feel distant from each other. Commonly, it was noted that the frequency of sex decreased, often leading to frustration or guilt on both sides. One study showed that proper communication, understanding each other’s situation, and spending time together led to an improvement in their relationship [26]. There were also positive changes among caregivers. For partners, parents, siblings, and a daughter of a patient, the diagnosis appeared to raise awareness and elicit healthier lifestyle adjustments, including paying more attention to their own wellbeing. A focus was placed on eating healthier, exercising more frequently, and participating in preventative health care. There was also more acceptance of vaccinations, sunscreen use, screening enrollment, and communication about health risks. In addition, there was more assertiveness toward health care professionals [27]. 

**Table 1:** Included articles on biological impact.

First Author [Ref.]	Country, Year	Study Design	Participant Characteristics (Age of Patient at Diagnosis)	Outcome Measures	Summary of Findings
Borstelmann [25]	USA, 2022	Cross-sectional	289 partners of AYA cancer patients (22 to 40 years old)	Sexuality (GMSEX)	20% of the participants reported sexual difficulties
Demiralp [18]	Turkey, 2010	Qualitative	2 spouses, 3 siblings, and 4 mothers of AYA cancer patients (16 to 38 years old)	Interviews to describe the personal experiences of family caregivers of patients with malignant tumors	Participants reported physical problems due to caregiving
Gorman [26]	USA, 2020	Qualitative	25 male partners of female AYA cancer patients (24 to 39 years old)	Interviews on how patients and partners appraise and manage their sexual health and intimate relationships after cancer	Participants reported a decrease in sexual health and different practices to improve this
Grinyer [24]	UK, 2006	Qualitative	9 mothers of AYA cancer patients (18 to 25 years old)	Interviews on the health of mothers and coping with these issues	Participants ignored their own physical health issues
Head [27]	USA, 2018	Qualitative	8 spouses, 5 mothers, 5 siblings, 2 partners, and 1 child of AYA cancer patients (18 to 36 years old)	Interviews to identify salient issues in relation to illness transformations for supporters	Participants report lifestyle changes and an altered attitude towards preventative healthcare
Iannarino [19]	USA, 2018	Qualitative	14 spouses, 6 siblings, 4 partners, and 1 ex-partner of AYA cancer survivors (18 to 39 years old)	Interview questions were designed to elicit narrative examples of participants’ experiences of biographical disruption, their attempts to navigate altered relationships, and their evaluations of others’ support attempts following biographical disruption	Participants were fatigued, which was caused by caregiving
Jeon [22]	Australia, 2020	Qualitative	3 caregivers of AYA cancer patients (aged 29 to 35 years old)	Explore sleep experiences of caregivers: perceptions of the nature and impact on sleep disturbances, potential ecological factors, and views on treatment options	Participants identified sleeping problems due to worrying and a lack of time to rest
Nolbris [23]	Sweden, 2014	Qualitative	9 siblings of AYA cancer patients (15 to 22 years old)	Interviews on the experiences of being a sibling of someone with cancer and narrating their memories and thoughts	Participants report difficulty sleeping during the night
Sanden [20]	Norway, 2008	Qualitative	A partner of an AYA cancer patient (27 years old)	Described the impact of living in a disrupted situation as partner to a patient with testicular cancer	Participant felt drained, leaving no energy for being emotional
Sari [21]	Turkey, 2013	Qualitative	13 parents of AYA cancer patients (15 to 17 years old)	Experiences of parents giving home care to their child on chemotherapy	Participants dealt with physical problems and were being hygienic to keep out viruses
Stevens [17]	UK, 2018	Mixed-methods	14 parents, 4 partners, 7 friends, and 4 other caregivers of AYA cancer patients (16 to 24 years old)	Unmet needs in cancer services	Participants reported impact on physical well-being due to caregiving

AYA, adolescent and young adult; GMSEX, Global Measure of Sexual Satisfaction.

### 3.2. Psychological Impact

Twenty-one studies examined the psychological impact of caregiving for an AYA cancer patient, including emotions, feelings, and positive changes (Table 2). Multiple studies report symptoms of post-traumatic stress disorder (PTSD) [28,29], depression [25,28,29,30,31,32], anxiety [18,23,25,28,29,30,33], and distress [28,34] among caregivers. Fear was a concept mentioned in many studies and included several components, e.g., fear of the patient dying [23,30,35,36,37], recurrence of the illness [32], metastasis [18], and whether the patient’s future will be different as their lives are disrupted in the early stages (career, friends, relationships, finances) [38,39]. A cancer diagnosis in someone close leads to various emotional changes in caregivers. Guilt is often prominent for caregivers, for reasons such as not being able to prevent the AYA from getting sick, or for being able to continue with their own life aside from taking care of the patient [19,26,30,35]. Additionally, anger [26,40], sadness [40], and feelings of grief [19,23,30] are often experienced by caregivers, as they watch as the patients have to give up their former lives and are often dissimilar from their peers. One young adult partner of a cancer patient described being so burned out that suicidal thoughts arose [19]. 

A lack of control over outcomes [18,36], an inability to unburden the patient, and managing the emotional state of the patient are all commonly reported challenges of a caregiver [37]. Many caregivers (i.e., parents, partners, and siblings) reported feeling helpless, especially when AYA cancer patients were distressed or let down due to consequences of the disease [19,35]. Similarly, caregivers found it challenging when they were not able to offer relief for the patient [23], and they had to observe the patient in pain or discomfort [30]. In addition, parents perceived injustice as their child was missing out on daily life activities, such as attending school or being with their peers [35], and questioned “Why is this happening to us?” [18]. The diagnosis also elicited shifts in how partners, parents, and siblings experienced life. These alterations included the realization of being able to die prematurely [27], and a life urgency in which they realized they could no longer wait to accomplish the things they desired [27,30]. 

Several studies found that partners, as well as parents, tended to put aside their own needs and fully devote themselves to caregiving [20,26,35,41]. They also tended not to show their worries and emotions to the patient or other family members, as there was a frequent dread of inciting the same fear or worry in others, as well [19,37]. Caregivers also struggled with what information to disclose to the patient, mainly if the patient was of a young age [35,42], and they had difficulty keeping their distance during healthcare visitations as they wanted the best for their patient [24,39]. Siblings often felt lonely and left out as parents had more focus on the patient and were often gone for long periods of time. At the same time, they tried to be understanding, deal with their own emotions and fears, and be a helpful source [23,37]. 

In addition to the negative outcomes of being a caregiver for a cancer patient, positive changes were frequently listed. The diagnosis often caused life to be re-appreciated. For example, there was an enhanced appreciation for the moments the siblings shared with the patient and their parents [37]. Additionally, parents realized what was important in their lives: happiness and health [36]. They redefined a ‘good day’ as one in which the patient was not feeling ill and was doing ‘okay’ [30]. Taking care of a cancer patient also entailed positive feelings: parents reported a sense of appreciation and compassion, while also recognizing the worth of everyday things [35]. Furthermore, taking care of an individual induced feelings of affection, determination, belonging, recognition, and happiness. Positive feedback from the AYA cancer patient increased caregivers’ confidence and willingness to perform the tasks [18].

**Table 2:** Included articles on psychological impact.

First Author [Ref.]	Country, Year	Study Design	Participant Characteristics (Age Patient at Diagnosis)	Outcome Measures	Summary of Findings
Baum [28]	USA, 2022	Cross-sectional	9 parents, 21 spouses, and 3 other caregivers of AYA cancer patients (16 to 39 years old)	PTSS (IES-R) Subjective distress (Distress Thermometer, Problem List) HRQoL (FACT-GP) Anxiety and depressive symptoms (ASR)	52% clinical PTSS 87% clinical distress 7% clinical depression 14% borderline depression 14% borderline anxiety 25% substance misuse Multiple factors related to PTSS
Bogetz [35]	USA, 2020	Qualitative	22 parents of AYA cancer patients (14 to 25 years old)	Interviews on communication, worries, information sharing, strengths, and support	Participants report many negative emotions and want to protect their AYA. They have difficulty balancing autonomy and advocating
Bogetz [40]	USA, 2022	Qualitative	22 parents of AYA cancer patients (14 to 25 years old)	Interviews on the process by which parents adapt to child’s serious illness	Participants experience negative emotions. There are also oscillating experiences during the disease, which they want to share
Borstelmann [25]	USA, 2022	Cross-sectional	289 partners of AYA cancer patients (22 to 40 years old)	Quality of life (CQOLC) Coping (BRIEF-COPE) Concerns (PCQ) Anxiety and Depression (HADS)	41% anxiety symptoms 18% depressive symptoms 51% parental concerns 44% maladaptive coping style
Cheng [31]	China, 2022	Cross-sectional	150 partners, 91 parents, 41 other (siblings, children or other relatives) caregivers of AYA cancer patients (15 to 39 years old)	Anxiety (GAD-7) Depression (PHQ-9) Quality of life (Quality of Life Family Scale)	79.4% mild and 39.4% moderate to severe anxiety 67.4% mild and 40.4% moderate to severe depression Impaired perceived quality of life Unmet needs are associated with psychological symptoms
Davies [41]	UK, 2019	Qualitative	3 partners of AYA cancer patients (19 to 20 years old)	Interviews on experiences of supporting an AYA with cancer	Participants reported to put themselves second and give up own comfort for caregiving
Demiralp [18]	Turkey, 2010	Qualitative	2 spouses, 3 siblings, and 4 mothers of AYA cancer patients (16 to 38 years old)	Interviews to describe the personal experiences of family caregivers of patients with malignant tumors	Participants identified various positive and negative emotions related to caregiving
Friesen [42]	Canada, 2002	Qualitative	4 children, 1 mother, 1 sibling, and 2 partners of 3 AYA cancer patients (28 to 40 years old)	Interviews regarding the impact of the disease on the family and their responses	Participants report difficulty in disclosing information to the patient
Gorman [26]	USA, 2020	Qualitative	25 male partners of female AYA cancer patients (24 to 39 years old)	Interviews on how patients and partners appraise and manage their sexual health and intimate relationships after cancer	Participants had negative emotions regarding reduced intimacy with their partner
Grinyer [24]	UK, 2006	Qualitative	9 mothers of AYA cancer patients (18 to 25 years old)	Interviews on the health of mothers coping with these issues	Participants reported difficulty balancing their input during hospital visits while being sensitive to independence
Head [27]	USA, 2018	Qualitative	8 spouses, 5 mothers, 5 siblings, 2 partners, and 1 child of AYA cancer patients (18 to 36 years old)	Interviews to identify salient issues related to illness transformations for supporters	Participants showed a changed outlook on life due to the disease
Iannarino [19]	USA, 2018	Qualitative	14 spouses, 6 siblings, 4 partners, and 1 ex-partner of AYA cancer survivors (18 to 39 years old)	Interview questions were designed to elicit narrative examples of participants’ experience of biographical disruption, their attempts to navigate altered relationships, and their evaluations of others’ support attempts following biographical disruption	Participants showed many negative emotions due to the disease and tended not to share those with others
McCarthy [29]	Australia, 2016	Cross-sectional	204 parent caretakers of AYA cancer patients (15 to 25 years old)	PTSS (PCL-S) Distress (K10) Impact of cancer (Life Impact Scale) Life stress	42% indication for PTSD 13% partial PTSD 28% moderate to severe depression or anxiety disorder Many factors related to psychological disorders
Mikrut [32]	USA, 2017	Cross-sectional	66 parents of AYA cancer patients (17 to 39 years old)	Social constraints on emotional disclosure (Lopre, 1999) Cognitive processing (IES-R) Fear of cancer recurrence (Concerns about Recurrence Scale) Depressive symptoms (PHQ-9)	33% had moderate to severe depressive symptoms Depressive symptoms were related to other negative psychological effects (for both cognitive processing and fear of recurrence: r = 0.57, significant at *p* < 0.01) Social constraint predicted more depressive symptoms
Mishra [30]	USA, 2018	Qualitative	5 partners, 1 parent, and 2 undefined caregivers of AYA cancer patients (20 to 39 years old)	Interviews to examine the experiences of cancer as an informal caregiver	Participants reported many negative psychological symptoms due to caregiving There were also positive changes in their outlook on life
Nam [34]	USA, 2016	Cross-sectional	Parents and other family caregivers of AYA cancer patients (15 to 21 years old)	Distress (IES) Sociodemographic and clinical variables	60.5% had a distress score of a clinically significant level (score above 26 on IES), which is an overall clinical concern for PTSD They had lower levels of intrusion compared to caregivers for younger patients (overall *p* = 0.02)
Nolbris [23]	Sweden, 2014	Qualitative	9 siblings of AYA cancer patients (15 to 22 years old)	Interviews on the experiences of being a sibling of someone with cancer, narrating their memories and thoughts	Participants experienced negative emotions because of the realization of mortality. They felt helpless and left out, and had to adjust to the situation
Palma [39]	USA, 2015	Qualitative	46 mothers of AYA patients (14 to 30 years old)	Identifying the daily maternal caregiver demands	Difficulty in letting the patient be independent during hospital visits, wanting to advocate
Sanden [20]	Norway, 2008	Case study	A partner of an AYA cancer patient (27 years old)	Described the impact of living in a disrupted situation as partner to a patient with testicular cancer	Participant put self second and neglected own needs. She wanted to experience everything together, but also has a changed future
Schweitzer [36]	Australia, 2014	Qualitative	2 parents from AYA cancer patients (15 to 17 years old)	Identifying the experiences related to the diagnosis	Diagnosis was a shock and they realized their child could die. Also, positive change in outlook on life occurred
Van Schoors [37]	Belgium, 2019	Qualitative	4 parents and three siblings of AYA cancer patients (16 years old)	Interviews on the experiences of the diagnostic and treatment process, as well as perspectives on family relationships and functioning post diagnosis	Participants felt separated from family. They had negative emotions but had a hard time talking about them. Also, felt like a team to take care of the patient.

ASR, Adult Self Report; AYA, Adolescent and Young Adult; CQOLC, Caregiver Quality of Life Index—Cancer; FACT-GP, Functional Assessment of Cancer Therapy—General Population; GAD, Generalized Anxiety Disorder; HADS, Hospital Anxiety and Depression; HRQoL, Health-Related Quality of Life; IES-R, Impact of Event Scale—Revised; K10, Kessler Psychological Distress Scale; PCL-S, PTSD Checklist; PCQ, Psychological Capital Questionnaire; PHQ, Patient Health Questionnaire; PTSD, Post-Traumatic Stress Disorder; PTSS, Post-Traumatic Stress Symptoms.

### 3.3. Social Impact

Thirteen studies reported on the social impact (i.e., relationships with others) of taking care of a young cancer patient (Table 3). The diagnosis impacted caregivers’ relationships and social activities. The study by Stevens (2018) reported that 17% of the networkers identified that the disease changed their relationship with the patient [17]. Support from others (e.g., healthcare professionals, parents having children with cancer, friends or family) was perceived as helpful to deal with the illness [18,20,40], where acknowledging the struggle and difficulty of the situation was supportive to the parents [40]. 

Several studies cite caregivers experiencing feelings of isolation and loneliness [19,21,24,40]. Caretaking caused them to be increasingly housebound and, as a result, their social life was impaired. Often, this was seen in mothers with caregiver tasks, while fathers mostly continued to be employed. This frequently resulted in a strain on relationships, as fathers did not want to talk about feelings and, instead, provided practical support for their child [24]. Partners of young breast cancer patients reported relationship strains with their sick partner due to the diagnosis (32%). They reported challenges with communicating with each other or discussing emotions [25]. A study conducted among parents of a child with cancer reported that those caring for a 15- to 19-year-old patient had a reduced probability of divorce and separation than those having younger children with cancer [43]. Additionally, there was no difference in the chance of separation between the caregivers of a child with cancer and a healthy child. The number of divorces was comparable, and was not related to the age of the offspring [33]. Caregivers sometimes reported that the disease positively impacted their relationship with the patient, as multiple studies identified the relationship between caregiver and patient as being strengthened [18,26,30,44].

Support from peers was considered as comforting, but given the young age of these peers, many often did not appear to have sufficient capabilities to be empathetic or understanding. Furthermore, fear of infection resulted in reduced social visits for family homes, which significantly impaired social interaction [21]. The longer post diagnosis, the less attention there was for the patient and caregiver. Additionally, a lot of social encounters concerned the patient, but not the caregiver. Some caregivers felt guilty towards the patient if they were able to manage a social life [19]. 

**Table 3:** Included articles on social impact.

First Author [Ref.]	Country, Year	Study Design	Participant Characteristics (Age of Patient at Diagnosis)	Outcome Measures	Summary of Findings
Bogetz [40]	USA, 2022	Qualitative	22 parents of AYA cancer patients (14 to 25 years old)	Interviews on the process by which parents adapt to child’s serious illness	Support from others was perceived as helpful Participants also felt isolated
Borstelmann [25]	USA, 2022	Cross-sectional	289 partners of AYA cancer patients (22 to 40 years old)	Relationship strain (CARES)	32% of all participants reported relationship strain
Demiralp [18]	Turkey, 2010	Qualitative	2 spouses, 3 siblings, and 4 mothers of AYA cancer patients (16 to 38 years old)	Interviews to describe the personal experiences of family caregivers of patients with malignant tumors	Participants experienced their family relationships as closer because of the disease
Gorman [26]	USA, 2020	Qualitative	25 male partners of female AYA cancer patients (24 to 39 years old)	Interviews on how patients and partners appraised and managed their sexual health and intimate relationships after cancer	Participants perceived a positive change in their relationship with their partner
Grinyer [24]	UK, 2006	Qualitative	9 mothers of AYA cancer patients (18 to 25 years old)	Interviews on the health of mothers coping with these issues	Participants felt lonely and experienced relationship strain due to different ways of coping Impaired social life
Iannarino [19]	USA, 2018	Qualitative	14 spouses, 6 siblings, 4 partners, and 1 ex-partner of AYA cancer survivors (18 to 39 years old)	Interview questions were designed to elicit narrative examples of participants’ experiences of biographical disruption, their attempts to navigate altered relationships, and their evaluations of others’ support attempts following biographical disruption	Participants experienced a lack of support from others. They also had difficult dynamics with other supporters
Mader [43]	Denmark, 2020	Cohort	2579 parents of AYA cancer patients (15 to 19 years old)	Separation, divorce, family planning, and sociodemographic and clinical data	Participants had a lower risk of divorce or separation compared to parents of younger children with cancer (HR > 1 for all age-groups below 15 years old)
Mishra [30]	USA, 2018	Qualitative	5 partners, 1 parent, and 2 undefined caregivers of AYA cancer patients (20 to 39 years old)	Interviews to examine the experiences of cancer for an informal caregiver	Participants identify stronger relationships to the patient due to the disease
Reblin [44]	USA, 2017	Qualitative	8 parents and 1 spouse of AYA cancer patients (19 to 29 years old)	Provided an insight into the expectations for types of psychosocial support using interviews	Participants identified that relationships became more meaningful Also, there were difficult dynamics between caregivers
Sanden [20]	Norway, 2008	Case study	A partner of an AYA cancer patient (27 years old)	Described the impact of living in a disrupted situation as partner to a patient with testicular cancer	Participant reported feeling a lot of support from family, which was perceived as helpful
Sari [21]	Turkey, 2013	Qualitative	13 parents of AYA cancer patients (15 to 17 years old)	Experiences of parents giving home care to their child on chemotherapy	Participants felt isolated as they reduced visits from others
Stevens [17]	UK, 2018	Mixed-method	14 parents, 4 partners, 7 friends, and 4 other caregivers of AYA cancer patients (16 to 24 years old)	Unmet needs in cancer services	Participants identified a changed relationship to the patient
Syse [33]	Norway, 2010	Cohort	Registry of all divorce rates in Norway with filter for AYA cancer patients (15 to 18 years old)	Divorce rates	Among participants there was no significant effect for divorce probability for being a parent of a child with or without cancer (OR 1.04, CI 0.95 to 1.13). Age of child with cancer did not have a significant effect (OR 1.01, CI 0.77 to 1.31)

AYA, adolescent and young adult; CARES, Cancer Rehabilitation Evaluation System Questionnaire.

### 3.4. Schedule Impact

Fourteen studies included information on the impact of caregiving on daily activities, the division of roles, and additional responsibilities (Table 4). It was reported that caregivers experienced challenges in maintaining daily tasks alongside performing caregiving duties for AYA cancer patients, as this was perceived as difficult and distressing [20,24,35]. Caregivers were frequently employed, which caused extra workload [19,44]. Parents described a challenging situation in which, on the one hand, they had to be parents, with all the rules and home duties that come with it, and on the other, they wanted to be responsive to the abnormal life the patient is leading and see their child happy [17,21,35,36]. Furthermore, partners reported difficulties in shifting from caregiver to sexual partner when the time came [26]. 

Activities of daily living were often shifted to the caregivers, including providing help with the washing, dressing, and transportation of the patient [18,21,39], administering drugs and food, managing and planning hospital visits, providing emotional support [17,19,20,21,39,44], and attending and advocating during visits to the healthcare professional [19,20,21,24,26,39]. Caregivers also assisted their patient in making difficult medical decisions [17,41], searched for specific information to pass on to the patient [19,42], took care of their children, as the patient often did not have the time or energy [42], and actively managed manifestations and signs of the disease [21,39]. In addition, mothers with caregiver duties helped to arrange plans for the AYAs’ future, such as for work, housing, or insurance [39]. Also, caregivers restrained others who tried to take away the independence of the AYA cancer patient by doing too much for them [19]. 

Caretakers also helped AYA cancer patients return to society during this process. For instance, partners helped them study, keeping them from dropping out of academia [41], as well as preparing them to return to their peers or colleagues by practicing conversations they may engage in or questions they may receive [42]. The responsibilities associated with caregiving can often result in reduced free time and availability for hobbies and relaxation [17,20,37]. Partners sacrificed their comforts at home (residence, sleeping spaces) to make the AYA as comfortable as possible [41]. They also experienced alterations in their future, where it may be more difficult to have children or to move house [19,20]. Parents applied hygiene rules and isolated themselves to minimize the risk of infection [21].

**Table 4:** Included articles on schedule impact.

First Author [Ref.]	Country, Year	Study Design	Participant Characteristics (Age of Patient at Diagnosis)	Outcome Measures	Summary of Findings
Bogetz [35]	USA, 2020	Qualitative	22 parents of AYA cancer patients (14 to 25 years old)	Interviews on communication, worries, information sharing, strengths, and support	Participants had difficulty with the role of being a parent. They also struggled with all roles and tasks they had to undertake
Davies [41]	UK, 2019	Qualitative	3 partners of AYA cancer patients (19 to 20 years old)	Interviews on experiences of supporting an AYA with cancer	Participants helped AYA to continue daily life. They worked more hours, moved closer to them and helped to make decisions
Demiralp [18]	Turkey, 2010	Qualitative	2 spouses, 3 siblings, and 4 mothers of AYA cancer patients (16 to 38 years old)	Interviews to describe the personal experiences of family caregivers of patients with malignant tumors	Participants helped the patient with their daily activities
Friesen [42]	Canada, 2002	Qualitative	4 children, 1 mother, 1 sibling, and 2 partners of 3 AYA cancer patients (28 to 40 years old)	Interviews regarding the impact of the disease on the family and their responses	Participants worked more hours to take care of AYA, took care of their families and helped them get back into society
Gorman [26]	USA, 2020	Qualitative	25 male partners of female AYA cancer patients (24 to 39 years old)	Interviews on how patients and partners appraise and manage their sexual health and intimate relationships after cancer	Participants joined AYA for hospital visits and advocated for them. Difficult to shift back to sexual partner
Grinyer [24]	UK, 2006	Qualitative	9 mothers of AYA cancer patients (18 to 25 years old)	Interviews on the health of mothers coping with these issues.	Caregivers reported many different roles and tasks
Iannarino [19]	USA, 2018	Qualitative	14 spouses, 6 siblings, 4 partners, and 1 ex-partner of AYA cancer survivors (18 to 39 years old)	Interview questions were designed to elicit narrative examples of participants’ experiences of biographical disruption, their attempts to navigate altered relationships, and their evaluations of others’ support attempts following biographical disruption	Caregivers took on many different roles and tasks in addition to their normative ones. Their own life plan was disrupted
Palma [39]	USA, 2015	Qualitative	46 mothers of AYA patients (14 to 30 years old)	Daily maternal caregiver demands	Participants had to manage all disease-related tasks and help with asking questions and making decisions. They advocated for them and helped them back into society
Reblin [44]	USA, 2017	Qualitative	8 parents and 1 spouse of AYA cancer patients (19 to 29 years old)	Provided insight into the expectations for types of psychosocial support using interviews	Participants were emotional support and assisted with travelling. Dividing roles were based on one’s characteristics, and it was difficult to undertake caregiving aside from normal activities
Sanden [20]	Norway, 2008	Case study	A partner of an AYA cancer patient (27 years old)	Described the impact of living in a disrupted situation as partner to a patient with testicular cancer	Participant managed medical care and emotional support and participated during healthcare visits. They gave up their own free time
Sari [21]	Turkey, 2013	Qualitative	13 parents of AYA cancer patients (15 to 17 years old)	Experiences of parents giving home care to their child on chemotherapy	Participants changed their living situation. They offered practical support and try to stay a parent in the meantime
Schweitzer [36]	Australia, 2014	Qualitative	2 parents of AYA cancer patients (15 to 17 years old)	Identifying the experiences related to the diagnosis	Participants felt like life revolved completely around hospital visits and medical care
Stevens [17]	UK, 2018	Mixed-method	14 parents, 4 partners, 7 friends, and 4 other caregivers of AYA cancer patients (16 to 24 years old)	Unmet needs in cancer services	Participants provided many types of support (emotional, practical, Advice, physical care, financial). Also, it was difficult to manage their normative tasks
Van Schoors [37]	Belgium,2019	Qualitative	4 parents and 3 siblings of AYA cancer patients (16 years old)	Interviews on the experiences of the diagnostic and treatment processes, as well as perspectives on family relationships and functioning post diagnosis	Participants gave up their own free time to take care of AYAs

AYA, adolescent and young adult.

### 3.5. Financial Impact

Ten studies reported a financial impact caused by caregiving for an AYA cancer patient (Table 5). AYAs with cancer are often too young and too sick to financially support themselves; as such, they rely on family members’ and caregivers’ support [45]. In multiple studies, financial problems were identified among the caregivers, particularly for the parents and partners [17,25,28,29,30,38,46]. The biggest expenses were due to travelling to the hospital and moving expenses [38,45], utilities, medical expenses (e.g., hairpieces, orthopedic materials, and particular nutrition or sanitization materials [38]), or mortgage repayments. Parking at the hospital was identified as the biggest cost for the majority of the participants [45]. 

Caregivers had come up with several solutions to their financial difficulties. When an AYA cancer patient was treated further away, partners and family caregivers accepted multiple jobs [38,42], worked additional hours [38,41], or changed jobs [38] to receive more income. Some caregivers relocated to live closer to the patient to provide support, leading to additional costs [19,41]. Parents also requested an allowance and used their saved income. Where possible, it was common to delay avoidable travelling and costs, as well as holidays. In addition, their own medical care was impaired, and caregivers would delay their own prescriptions or wait for general medication to be accessible [38]. The loss of income was greater if the patient was still living with parents and if the parents were self-employed [45]. Anxiety and stress occurred due to unforeseen expenses, as well as sudden appointments, medication, or medical procedures. Parents often tried to protect AYA cancer patients from the impact their diagnosis had on finances by covering up bills, not disclosing the impact, and reassuring them. They were afraid the patients would be stressed or ‘crushed’ if they knew [38]. 

**Table 5:** Included studies on financial impact.

First Author [Ref.]	Country, Year	Study Design	Participant Characteristics (Age of Patient at Diagnosis)	Outcome Measures	Summary of Findings
Baum [28]	USA, 2022	Cross-sectional	9 parents, 21 spouses, and 3 other caregivers of AYA cancer patients (16 to 39 years old)	Financial toxicity (Cost)	43.9% of participants had financial toxicity
Borstelmann [25]	USA, 2022	Cross-sectional	289 partners of AYA cancer patients (22 to 40 years old)	Financial security (self-developed)	29% of participants had financial insecurity
Davies [41]	UK, 2019	Qualitative	3 partners of AYA cancer patients (19 to 20 years old)	Interviews on experiences of supporting an AYA with cancer	Moving closer to the patient increased costs for participants
Friesen [42]	Canada, 2002	Qualitative	4 children, 1 mother, 1 sibling, and 2 partners of 3 AYA cancer patients (28 to 40 years old)	Interviews regarding the impact of the disease on the family, and their responses	Participants took on multiple jobs to increase income
Iannarino [19]	USA, 2018	Qualitative	14 spouses, 6 siblings, 4 partners, and 1 ex-partner of AYA cancer survivors (18 to 39 years old)	Interview questions were designed to elicit narrative examples of participants’ experience of biographical disruption, their attempts to navigate altered relationships, and their evaluations of others’ support attempts following biographical disruption	Participants moved closer to the patient, leading to additional costs
McCarthy [46]	Australia, 2018	Cross-sectional	204 parent caretakers of AYA cancer patients (15 to 25 years old)	Information needs	47% of participants needed financial support after treatment
McNeil [45]	Finland, 2019	Cross-sectional	204 parent caretakers of AYA cancer patients (15 to 25 years old)	Self-developed questionnaire on financial impact Use of income support	62% of participants had financial difficulties and 38% wanted support for this during treatment. 68% also wanted this after treatment. 32% had problems in receiving this support
Mishra [30]	USA, 2018	Qualitative	5 partners, 1 parent, and 2 undefined caregivers of AYA cancer patients (20 to 39 years old)	Interviews to examine the experiences of cancer as an informal caregiver	Participants reported financial burdens due to the disease
Nightingale [38]	USA, 2021	Qualitative	23 parents and 1 grandparent of AYA cancer patients (15 to 39 years old)	Interviews eliciting understanding and experiences of financial aspects of cancer	Participants reported many financial issues due to disease-related expenses. They shielded their AYA from this burden
Stevens [17]	UK, 2018	Mixed-method	14 parents, 4 partners, 7 friends, and 4 other caregivers of AYA cancer patients (16 to 24 years old)	Unmet needs in cancer services	Participants reported financial problems due to the disease

AYA, adolescent and young adult; Cost, Comprehensive Score for Financial Toxicity.

### 3.6. Unmet Needs

Fifteen studies identified the unmet needs of caregivers of AYA cancer patients during the disease phases, and subsequently identified their coping methods (Table 6). When obtaining information about the cancer diagnosis, it was reported that caregivers would like to receive this both verbally and written on paper [17,30,38]. According to caregivers, information was lacking regarding the cancer itself, which could help them understand information regarding the therapy and effects of the treatment [30,31,46], the prognosis, the cancer returning or the disease worsening [18,31,46], and alternative or supplementary treatment options [31,46], which helps them to make decisions. Parents preferred visual information, such as diagrams [30].

Caregivers also reported that they would benefit from talking about the cancer, and its related consequences and emotions also appeared to be something for which help was wanted. Caregivers sought support in discussing sensitive topics with the patients (e.g., details of their diagnosis), peer support [46], patients’ intimacy and sexuality [24,46], and emotions and feelings [46]. The caregivers themselves also wanted the opportunity to talk about their own experiences related to the disease (emotional counseling or talking to a mental healthcare professional) [17,47], as well as spirituality or religion in light of the disease [47], and support from others going through the same process [46], and to have a chance to share what life is really like as a caregiver of a young person with cancer [40]. In addition, they would like assistance with practical issues, such as managing their finances [17,38,45,46,47], properly performing caregiving tasks for their AYA [26,46], and coping with sleep difficulties [22], and support in making important (medical) choices [17,30]. Caregivers would like to see this information adjusted to their age or situation. The information should be understandable for those of younger ages, who are often not familiar with the healthcare system [19]. 

Caregivers reported various strategies to cope with the stress of the disease. They tended to take it one day at a time [30,35,40] in order to alleviate their worrying and remain in control. Some caregivers turned to religion to cope with the disease [18,30,35,40]. Additionally, parents used time off, physical activity, narrative writing, or communication with others as coping strategies. Other parents did not speak about the disease, and preferred to be alone to cope with their emotions. Meditation and mindfulness were reported as ways of coping by family caregivers, as this reduced stress [22]. Some caregivers distracted themselves from thoughts relating to the disease through means such as alcohol consumption [30]. Hodgson et al. (2021) identified that the coping styles of patients and caregivers were related, and that when one was coping well, the other experienced less stress [48].

**Table 6:** Included studies on unmet needs.

First Author [Ref.]	Country, Year	Study Design	Participant Characteristics (Age of Patient at Diagnosis)	Outcome Measures	Summary of Findings
Bogetz [35]	USA, 2020	Qualitative	22 parents of AYA cancer patients (14 to 25 years old)	Interviews on communication, worries, information sharing, strengths and support	Participants reported various ways to cope with the disease
Bogetz [40]	USA, 2022	Qualitative	22 parents of AYA cancer patients (14 to 25 years old)	Interviews on the processes by which parents adapted to child’s serious illness	Participants had preferences for support and felt the need to share their experience. They reported various ways of coping
Cheng [31]	China, 2022	Cross-sectional	150 partners, 91 parents, and 41 other (siblings, children, or other relatives) caregivers of AYA cancer patients (15 to 39 years old)	Unmet needs (SPUNS-SFC)	Of the participants, at least 98.9% had one unmet need. Needs were positively associated with anxiety and depression
Demiralp [18]	Turkey, 2010	Qualitative	2 spouses, 3 siblings, and 4 mothers of AYA cancer patients (16 to 38 years old)	Interviews to describe the personal experiences of family caregivers of patients with malignant tumors	Participants reported various ways to cope with the disease
Gorman [26]	USA, 2020	Qualitative	25 male partners of female AYA cancer patients (24 to 39 years old)	Interviews on how patients and partners appraised and managed their sexual health and intimate relationships after cancer	Participants reported a preference for support for their sexual health
Grinyer [24]	UK, 2006	Qualitative	9 mothers of AYA cancer patients (18 to 25 years old)	Interviews on the health of mothers coping with these issues	Participants wished for support on how to talk to the patient regarding sexuality
Hodgson [48]	USA, 2021	Cross-sectional	5 parents, 2 partners, 10 spouses, and two other family members of AYA cancer patients (18 to 41 years old)	Distress (Distress Thermometer) Patient problems (Patient Problems List) Coping (Coping Strategies Inventory—Short Forms)	In participants, adaptive coping was related to less distress (*B* = −0.137, *p* < 0.05). An engaging coping style from patients was related to fewer patient problems for caregivers (*B* = −0.489, *p* = 0.014)
Iannarino [19]	USA, 2018	Qualitative	14 spouses, 6 siblings, 4 partners, and 1 ex-partner of AYA cancer survivors (18 to 39 years old)	Interview questions were designed to elicit narrative examples of participants’ experiences of biographical disruption, their attempts to navigate altered relationships, and their evaluations of others’ support attempts following biographical disruption	Participants reported a need for understandable information for all ages and those not familiar with the healthcare system
Jeon [22]	Australia, 2020	Qualitative	3 caregivers of AYA cancer patients (aged 29 to 35 years old)	Explored sleep experiences of caregivers: perceptions of the nature and impact on sleep disturbances, potential ecological factors, and views on treatment options	Participants reported various ways to cope with the disease
McCarthy [46]	Australia, 2018	Cross-sectional	204 parent caretakers of AYA cancer patients (15 to 25 years old)	Information needs Patient activation (PAM)	50% of participants had information needs in any domain. 30% had support needs for themselves and the patient. They wanted to be able to discuss with the patient what is happening
McNeil [45]	Finland, 2019	Cross-sectional	204 parent caretakers of AYA cancer patients (15 to 25 years old)	Self-developed questionnaire on financial impact Use of income support Education and work impact Demographics and clinical variables	38% wanted income support during treatment. 68% of them also wanted this support after treatment completion. 32% had difficulty receiving financial support due to multiple reasons
Mishra [30]	USA, 2018	Qualitative	5 partners, 1 parent, and 2 undefined caregivers of AYA cancer patients (20 to 39 years old)	Interviews to examine the experiences of cancer for an informal caregiver	Participants reported various ways of coping. They had a need for information on multiple domains
Nightingale [38]	USA, 2021	Qualitative	23 parents and 1 grandparent of AYA cancer patients (15 to 39 years old)	Interviews eliciting understanding and experiences of financial aspects of cancer	Participants had a need for financial support and information. They had many ways to cope with the financial issues
Sawyer [47]	Australia, 2017	Cross-sectional	204 parents of AYA cancer patients (15 to 25 years old)	Service needed (AYA Hope/CNQ-PC) Post-traumatic stress (PCL-S)	Participants had a need to talk to a social worker, and wanted a peer support group and religious counselling
Stevens [17]	UK, 2018	Mixed-method	14 parents, 4 partners, 7 friends, and 4 other caregivers of AYA cancer patients (16 to 24 years old)	Unmet needs in cancer services	There was a need for support in all domains of life among these participants. They wanted face-to-face guidance from the hospital

AYA, adolescent and young adult; CNQ-PC, Cancer Needs Questionnaire Parents Carers; PAM, Patient Activation Measure; SPUNS-SFC, Cancer Support Person’s Unmet Needs Survey—Short Form.

## 4. Discussion

**Figure 2:**
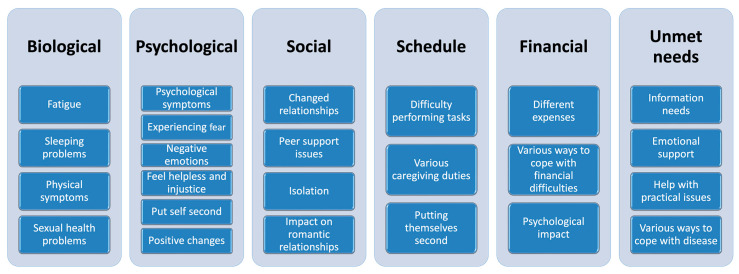
Overview of themes within different domains of caregiver burden.

This review provided an overview of the impact of being a caregiver for an AYA cancer patient on different domains of daily life, including biological, psychological, social, schedule, financial, and unmet needs (Figure 2). Due to the lack of quantitative data and all the various caregivers caring for AYA cancer patients due to the wide age-range, it is difficult to make firm statements on what defines these caregivers. Among the many reported burdens caregivers experience, there are certain outcomes that could be classified as age-specific and arise from the life stage of the patient. These caregiver challenges are mainly related to the emotions centered around having to give up a normal life at such a young age, where the possibility of a changed future for the AYA cancer patient also leads to concern. In addition, it is often perceived as challenging to determine which role the caregivers should take, as they often hold multiple roles during the course of an AYA’s disease. For young caregivers, it is challenging that their peers cannot empathize with the experience and therefore cannot provide appropriate support. Furthermore, for young partners, their own future is also affected by the patient’s illness and they must deal with these alterations. Caregivers also report information and emotional needs.

Parents are more likely to be involved with younger patients, and are therefore more burdened when the patient is younger. It is common for parents to worry about their child’s future, in terms of academia and career prospects, and to struggle with the realization that their child is not living a traditional AYA lifestyle. Parents may isolate themselves from others during the treatment to reduce the possibility that their child will contract an infection, which directly impacts their social interaction. They also carry the financial burden of their child’s disease. In the case of young adolescents, parents struggle with which information to disclose to their child, and do not share the emotional impact of it as they are frightened of worrying their child or other family members. The literature reported difficulties for parents of adolescents to determine what their role was, for example, when it comes to making decisions. In some cases, the child is capable of making decisions independently, or their child may have a different opinion, leading to conflict [49]. To date, there are few articles that focus on the impact of parents of young cancer patients and possible interventions to support them. Parents of adolescents with cancer did report that being in the hospital, together with others dealing with the same disease, was perceived as helpful and supportive [49]. Thus, setting up support groups including parents of adolescents with cancer could be adequate care for these parents. In pediatric oncology, more psychosocial support and interventions are available than in the AYA age-group. Here, cognitive behavioral therapy is often recommended, reducing distress levels, depression, and anxiety. It can also help to improve parents’ capabilities for solving issues [50]. Optionally, this type of psychological treatment could also be supportive for adolescent parents.

Partners of AYA cancer patients experience major consequences in their daily functioning and future plans. From a young age, their lives revolve around hospital visits and providing care, while prior to that they were occupied with studying, work, or seeing their friends. In addition, the decision to start a family or to make other major life choices (such as moving house) may be difficult. A study by Hoellen et al. (2019) showed that partners younger than 35 years old report more anxiety than older partners. This discrepancy is partly caused by fears regarding the future, the desire to start a family and pursuing household tasks during their partners’ disease (i.e., age-related topics). This fear impairs their ability to provide proper support, and the authors recommended psychological help for partners [51]. The current review also identified that permanent fertility problems of the patient can lead to difficulties in raising a family. Partners additionally recognized the impact on their sexual health and activity due to two reasons: 1) the division of roles becoming different within the relationship, and 2) the physical conditions of the patient. Interestingly, there were no interventions tailored to young partners of cancer patients. Jones and colleagues (2013) studied a group intervention for partners, which aimed to have the partners acknowledge the impact of the disease on their lives, while also scheduling moments to take for themselves throughout the day. In addition, suboptimal communication with their sick partner was recognized, and therefore techniques were developed to improve this. Focus was placed on their ill partners’ needs and how to provide sufficient support, and at the same time how to distance themselves from this whenever feasible. This intervention reduced anxiety, improved social support, and resulted in partners being able to take care of themselves, not ignoring their own needs [52], which was described as a consequence of caregiving in many articles in this review.

Siblings of AYA cancer patients reported feeling lonely during the disease trajectory, mainly when they were of a young age. Parents spent a lot of time with the patient, and both were frequently away from home. Siblings were found to actively avoid over-burdening their parents by minimizing asking for attention or sharing their emotions with them. However, there are few works in the literature on siblings of AYA cancer patients and interventions specifically for older siblings. The literature mainly focused on siblings of pediatric cancer patients, for whom the impact on their daily lives was perceived as greater. This is due to the fact that they still live with the patient. Nonetheless, the strength of the relationship between patient and sibling was also of significance for the impact on their life. For siblings of pediatric patients, adventure camps are often organized, in which peer support is the main focus [53,54,55]. Social interactions with others dealing with the same challenges can diminish the loneliness they are experiencing. In addition, being away from home can give room for emotions and challenges to be shared without the impression of having to protect family members’ feelings. Meeting peers can also normalize their daily life struggles [53]. This intervention could possibly be implemented for younger siblings of AYA cancer patients. The current review showed that worrying also occurred frequently, and could cause psychological symptoms (e.g., distress) and difficulty sleeping. In these articles, information on the disease and current events were mentioned to cause a reduction in worrying. A study by Northouse and colleagues (2012) on adult family caregivers identified multiple strategies leading to better health outcomes for both patients and caregivers. They recommended expressing feelings and concerns, which can reduce stress and help both siblings and patients to resolve problems together. Being able to listen and show understanding is helpful. Gathering information, as well as attending hospital appointments, helps to gain insight into the disease and confidence in one’s own abilities, while also removing uncertainty. Where necessary, additional help is recommended [56]. In this case, cognitive-behavioral interventions for family members of cancer patients help to reduce difficulty sleeping and distress, and to improve adaptations to the new situation [57].

Studies included in this review also focused on the unmet needs of caregivers of AYA cancer patients, which could be used to develop appropriate interventions. There was a demand for more information regarding different aspects of the disease, making caregivers feel prepared for their tasks and giving them the perception that they had knowledge and control. A review by Bevan and Pecchioni (2008) suggested adapting the information to the specific caregiver and their literacy level. In addition, they advised educating peers with information, so they could also inform the patient and help with decision making, lowering the psychological burden on the (informal) caregiver. This article also stressed the need for written communication regarding the disease shared by the health care professional [58]. The current review also reported the need for caregivers to talk to others about the disease, as well as support in talking to the patient about it. As the outcomes for patients and caregivers are related, dyadic interventions could be a valuable option. This type of intervention is linked to increased emotional, social, spiritual, and psychological domains of quality of life, while also improving the connection between the caregiver and patient. Interventions that were frequently applied provided information and improved caregiving capabilities, whilst also focusing on the relationship between patient and caregiver, and on skills for coping and social support [59]. 

This review contributes to the awareness regarding the AYA cancer caregiver burden. However, this study is also subject to limitations. The works from the literature used in this review were largely qualitative in nature. This means that their interpretation depends on the data used. In addition, some studies reported only limited information on caregivers of AYA cancer patients, and sometimes there were restricted data available. In this respect, the influence on the caregiver sometimes appeared to be very large, but was only reported in small numbers. Caregiver impact is a vague term, and no clear definition was used during the literature search. This might have led to some bias because of missing studies. No studies were included that did not specify a defined age limit for the patients being cared for. This means that some articles reporting some information about caregivers of AYA cancer patients may have been excluded. The difference in caregivers (e.g., the role of parents) between older and younger (15–25 years old) AYAs was underrepresented in this article, but may be a significant factor in the different impacts discussed.

Future research on informal caregivers of AYA cancer patients could explore the needs of different types of caregivers in greater depth, with more focus on what age-specific issues and needs exist for each group. This information will help to develop support that is more specific for each type of caregiver. Currently, there are few specific interventions for caregivers in the literature. In addition, several groups seem understudied and require further research; more research has been undertaken on mothers than fathers, and there are few works in the literature on caregivers who do not belong to family circles, such as friends. Additionally, many studies base their results on small samples. It is also important to examine the underlying dynamics between caregiver and patient in more depth. In the absence of parents, caregiving falls to the responsibility of different individuals. Some relationships with others (e.g., friends or a romantic partner) deteriorate because of the disease, but often this impact is shielded from the patient to avoid imposing an additional burden on them. Some relationships, on the other hand, do end because of the disease. Follow-up research is needed to test the current findings with larger groups of caregivers of AYA cancer patients in order to better define age-specific complaints and needs and to make it possible to distinguish between certain groups with certain problems or needs. In addition, the age-range of AYAs is large, and many different caregivers are involved as a result. Research is needed to see which individuals take part, for how long they stay involved, and what changes occur in the relationship due to the patient’s disease. Appropriate interventions or care can be considered, subsequently, as well as who will be responsible for conducting this specific care.

## 5. Conclusions

In conclusion, it can be argued that caregiving for an AYA cancer patient entails a large burden that includes different domains of life, e.g., biological, psychological, social, schedule, and financial. There are needs that have to be fulfilled to support them. By being more attentive to the caregivers and their needs, and intervening accordingly (e.g., social support or psychological interventions), caregivers’ quality of life can increase, which allows for better caregiving capabilities. This not only has a positive impact on the patient, but it might also lead to decreased caregiver burden.

## Supplementary Materials

The following supporting information can be downloaded at: https://www.mdpi.com/article/10.3390/cancers15123263/s1, Data S1: PRISMA-checklist for scoping reviews; Data S2: Search strategy for the scoping review.
