# Supplementary material for: Caring for Adolescents and Young Adults (AYA) with Cancer: A Scoping Review into Caregiver Burdens and Needs
Source: Cancers (Basel). 2023 Jun 20;15(12):3263. doi: 10.3390/cancers15123263 (PMC10297107; doi:10.3390/cancers15123263)
Supplement: Supplementary file 1 [file cancers-15-03263-s001.zip › cancers-2391873-supplementary.pdf]

Data S1. PRISMA-checklist for scoping reviews

Section and Topic	Item #	Checklist item	Location where item is reported
**TITLE**			
Title	1	Identify the report as a scoping review.	Page 1
**ABSTRACT**			
Abstract	2	See the PRISMA 2020 for Abstracts checklist.	Checked
**INTRODUCTION**			
Rationale	3	Describe the rationale for the review in the context of existing knowledge.	Page 1-3
Objectives	4	Provide an explicit statement of the objective(s) or question(s) the review addresses.	Page 3
**METHODS**			
Eligibility criteria	5	Specify the inclusion and exclusion criteria for the review and how studies were grouped for the syntheses.	Page 3
Information sources	6	Specify all databases, registers, websites, organisations, reference lists and other sources searched or consulted to identify studies. Specify the date when each source was last searched or consulted.	Page 3
Search strategy	7	Present the full search strategies for all databases, registers and websites, including any filters and limits used.	Appendix
Selection process	8	Specify the methods used to decide whether a study met the inclusion criteria of the review, including how many reviewers screened each record and each report retrieved, whether they worked independently, and if applicable, details of automation tools used in the process.	Page 4
Data collection process	9	Specify the methods used to collect data from reports, including how many reviewers collected data from each report, whether they worked independently, any processes for obtaining or confirming data from study investigators, and if applicable, details of automation tools used in the process.	Page 3-4
Data items	10a	List and define all outcomes for which data were sought. Specify whether all results that were compatible with each outcome domain in each study were sought (e.g. for all measures, time points, analyses), and if not, the methods used to decide which results to collect.	Page 3-4
	10b	List and define all other variables for which data were sought (e.g. participant and intervention characteristics, funding sources). Describe any assumptions made about any missing or unclear information.	Page 3-4
Study risk of bias assessment	11	Specify the methods used to assess risk of bias in the included studies, including details of the tool(s) used, how many reviewers assessed each study and whether they worked independently, and if applicable, details of automation tools used in the process.	Page 4
Effect measures	12	Specify for each outcome the effect measure(s) (e.g. risk ratio, mean difference) used in the synthesis or presentation of results.	N/A
Synthesis methods	13a	Describe the processes used to decide which studies were eligible for each synthesis (e.g. tabulating the study intervention characteristics and comparing against the planned groups for each synthesis (item #5)).	Page 3-4

Section and Topic	Item #	Checklist item	Location where item is reported
	13b	Describe any methods required to prepare the data for presentation or synthesis, such as handling of missing summary statistics, or data conversions.	N/A
	13c	Describe any methods used to tabulate or visually display results of individual studies and syntheses.	N/A
	13d	Describe any methods used to synthesize results and provide a rationale for the choice(s). If meta-analysis was performed, describe the model(s), method(s) to identify the presence and extent of statistical heterogeneity, and software package(s) used.	N/A
	13e	Describe any methods used to explore possible causes of heterogeneity among study results (e.g. subgroup analysis, meta-regression).	N/A
	13f	Describe any sensitivity analyses conducted to assess robustness of the synthesized results.	N/A
Reporting bias assessment	14	Describe any methods used to assess risk of bias due to missing results in a synthesis (arising from reporting biases).	N/A
Certainty assessment	15	Describe any methods used to assess certainty (or confidence) in the body of evidence for an outcome.	N/A
**RESULTS**			
Study selection	16a	Describe the results of the search and selection process, from the number of records identified in the search to the number of studies included in the review, ideally using a flow diagram.	Page 4
	16b	Cite studies that might appear to meet the inclusion criteria, but which were excluded, and explain why they were excluded.	Page 4
Study characteristics	17	Cite each included study and present its characteristics.	Page 5-19
Risk of bias in studies	18	Present assessments of risk of bias for each included study.	N/A
Results of individual studies	19	For all outcomes, present, for each study: (a) summary statistics for each group (where appropriate) and (b) an effect estimate and its precision (e.g. confidence/credible interval), ideally using structured tables or plots.	N/A
Results of syntheses	20a	For each synthesis, briefly summarise the characteristics and risk of bias among contributing studies.	Page 5-19
	20b	Present results of all statistical syntheses conducted. If meta-analysis was done, present for each the summary estimate and its precision (e.g. confidence/credible interval) and measures of statistical heterogeneity. If comparing groups, describe the direction of the effect.	N/A
	20c	Present results of all investigations of possible causes of heterogeneity among study results.	N/A
	20d	Present results of all sensitivity analyses conducted to assess the robustness of the synthesized results.	N/A
Reporting biases	21	Present assessments of risk of bias due to missing results (arising from reporting biases) for each synthesis assessed.	N/A

Section and Topic	Item #	Checklist item	Location where item is reported
Certainty of evidence	22	Present assessments of certainty (or confidence) in the body of evidence for each outcome assessed.	N/A
**DISCUSSION**			
Discussion	23a	Provide a general interpretation of the results in the context of other evidence.	Page 20-22
	23b	Discuss any limitations of the evidence included in the review.	Page 22
	23c	Discuss any limitations of the review processes used.	Page 22
	23d	Discuss implications of the results for practice, policy, and future research.	Page 22
**OTHER INFORMATION**			
Registration and protocol	24a	Provide registration information for the review, including register name and registration number, or state that the review was not registered.	Page 22
	24b	Indicate where the review protocol can be accessed, or state that a protocol was not prepared.	Page 22
	24c	Describe and explain any amendments to information provided at registration or in the protocol.	N/A
Support	25	Describe sources of financial or non-financial support for the review, and the role of the funders or sponsors in the review.	Page 22
Competing interests	26	Declare any competing interests of review authors.	Page 22
Availability of data, code and other materials	27	Report which of the following are publicly available and where they can be found: template data collection forms; data extracted from included studies; data used for all analyses; analytic code; any other materials used in the review.	N/A

Data S2. Search strategy for the scoping review

Search History:

2022-10-28

Ovid MEDLINE(R) ALL <1946 to October 27, 2022>

#	Query	Hits
1	adolescent/	2191794
2	"young adult"/	999023
3	adolescent*.ti,ab,kf.	310178
4	"young adult*".ti,ab,kf.	115611
5	teen.ti,ab,kf.	6308
6	teenage*.ti,ab,kf.	24048
7	AYA.ti,ab,kf.	2117
8	AYAs.ti,ab,kf.	1241
9	TYA?.ti,ab,kf.	617
10	"emerging adult*".ti,ab,kf.	3819
11	or/1-10 [AYAs]	2775425
12	exp neoplasms/	3750717
13	neoplas*.ti,ab,kf.	439092
14	tumo?r?.ti,ab,kf.	1914552
15	cancer?.ti,ab,kf.	2139381
16	malignan*.ti,ab,kf.	660980
17	metasta*.ti,ab,kf.	599011
18	carcinoma?.ti,ab,kf.	743589
19	melanoma?.ti,ab,kf.	133943
20	sarcoma?.ti,ab,kf.	104479
21	leuk?emia?.ti,ab,kf.	281750
22	lymphoma?.ti,ab,kf.	198622
23	or/12-22 [cancer]	4986597
24	caregiver?.ti,ab,kf.	84440
25	"care giver?".ti,ab,kf.	2941
26	(informal adj3 (care* or support*)).ti,ab,kf.	9664
27	((family or families or spous* or partner? or parent* or sibling? or friend? or relative? or "loved one?") adj3 (carer* or support*)).ti,ab,kf.	45859
28	(informal or family or families or spous* or partner? or parent* or sibling? or friend? or relative? or "loved one?").ti,ab,kf. and caregiver/	29393
29	"caregiver burden"/	487
30	(care* adj3 (burden or needs)).ti,ab,kf.	40236
31	or/24-30 [informal caregivers]	163399
32	"caregiver burden"/	487
33	"needs assessment"/	32260
34	burden*.ti,ab,kf.	295410
35	impact*.ti,ab,kf.	1451556
36	needs.ti,ab,kf.	406577
37	experience*.ti,ab,kf.	1285780
38	support.ti,ab,kf.	1197195
39	or/32-38 [burden]	4055126
40	11 and 23 and 31 and 39 [AYAs AND cancer AND informal caregivers AND burden]	2190

Single line search:

((adolescent/ OR "young adult"/) OR (adolescent* OR "young adult*" OR teen OR teenage* OR AYA OR AYAs OR TYA? OR "emerging adult*").ti,ab,kf.) AND (exp neoplasms/ OR (neoplas* OR tumo?r? OR cancer? OR malignan* OR metasta* OR carcinoma? OR melanoma? OR sarcoma? OR leuk?emia? OR lymphoma?).ti,ab,kf.) AND ((caregiver? OR "care giver?").ti,ab,kf. OR (informal ADJ3 (care* OR support*)).ti,ab,kf. OR ((family OR families OR spous* OR partner? OR parent* OR sibling? OR friend? OR relative? OR "loved one?") ADJ3 (carer* OR support*)).ti,ab,kf. OR ((informal OR family OR families OR spous* OR partner? OR parent* OR sibling? OR friend? OR relative? OR "loved one?").ti,ab,kf. AND caregiver/) OR "caregiver burden"/ OR (care* ADJ3 (burden OR needs)).ti,ab,kf.) AND (("caregiver burden"/ OR "needs assessment"/) OR (burden* OR impact* OR needs OR experience* OR support).ti,ab,kf.)

Embase.com:

#	**Query**	**Hits**
1	'adolescent'/exp	1857682
2	'young adult'/exp	478293
3	adolescent*:ti,ab,kw	409701
4	'young adult*':ti,ab,kw	153692
5	teen:ti,ab,kw	8394
6	teenage*:ti,ab,kw	34172
7	aya$:ti,ab,kw	4821
8	tya$:ti,ab,kw	369
9	'emerging adult*':ti,ab,kw	4305
10	#1 OR #2 OR #3 OR #4 OR #5 OR #6 OR #7 OR #8 OR #9	2328942
11	'neoplasm'/exp	5789748
12	neoplas*:ti,ab,kw	530858
13	tumo$r$:ti,ab,kw	2635304
14	cancer$:ti,ab,kw	3067846
15	malignan*:ti,ab,kw	955136
16	metasta*:ti,ab,kw	877937
17	carcinoma$:ti,ab,kw	1015647
18	melanoma$:ti,ab,kw	189928
19	sarcoma$:ti,ab,kw	130960
20	leuk$emia$:ti,ab,kw	395899
21	lymphoma$:ti,ab,kw	294715
22	#11 OR #12 OR #13 OR #14 OR #15 OR #16 OR #17 OR #18 OR #19 OR #20 OR #21	6894756
23	'informal care'/de	59
24	'informal caregiver'/de	46
25	caregiver$:ti,ab,kw	118371
26	'care giver$':ti,ab,kw	5456
27	(informal NEAR/3 (care* OR support*)):ti,ab,kw	11943
28	((family OR families OR spous* OR partner$ OR parent* OR sibling$ OR friend$ OR relative$ OR 'loved one$') NEAR/3 (carer* OR support*)):ti,ab,kw	60301
29	(informal:ti,ab,kw OR family:ti,ab,kw OR families:ti,ab,kw OR spous*:ti,ab,kw OR partner$:ti,ab,kw OR parent*:ti,ab,kw OR sibling$:ti,ab,kw OR friend$:ti,ab,kw OR relative*:ti,ab,kw OR 'loved one$':ti,ab,kw) AND 'caregiver'/de	54850
30	'caregiver burden'/de	9818
31	(care* NEAR/3 (burden OR needs)):ti,ab,kw	56285
32	#23 OR #24 OR #25 OR #26 OR #27 OR #28 OR #29 OR #30 OR #31	223861

33	'caregiver burden'/de	9818
34	burden*:ti,ab,kw	449855
35	impact*:ti,ab,kw	2026017
36	needs:ti,ab,kw	554317
37	experience*:ti,ab,kw	1828517
38	support:ti,ab,kw	1548000
39	#33 OR #34 OR #35 OR #36 OR #37 OR #38	5523487
40	#10 AND #22 AND #32 AND #39	2541
41	#40 NOT ('conference abstract'/it OR 'conference paper'/it OR 'conference review'/it)	1787
42	#40 AND ('conference abstract'/it OR 'conference paper'/it OR 'conference review'/it)	754

Single line search:
((adolescent/exp OR 'young adult'/exp) OR (adolescent* OR 'young adult*' OR teen OR teenage* OR AYA$ OR TYA$ OR 'emerging adult*'):ti,ab,kw) AND (neoplasm/exp OR (neoplas* OR tumo$r$ OR cancer$ OR malignan* OR metasta* OR carcinoma$ OR melanoma$ OR sarcoma$ OR leuk$emia$ OR lymphoma$):ti,ab,kw) AND (('informal care'/de OR 'informal caregiver'/de OR 'informal caregiving'/de) OR (caregiver$ OR 'care giver$'):ti,ab,kw OR (informal NEAR/3 (care* OR support*)):ti,ab,kw OR ((family OR families OR spous* OR partner$ OR parent* OR sibling$ OR friend$ OR relative$ OR 'loved one$') NEAR/3 (carer* OR support*)):ti,ab,kw OR ((informal OR family OR families OR spous* OR partner$ OR parent* OR sibling$ OR friend$ OR relative* OR 'loved one$'):ti,ab,kw AND caregiver/de) OR 'caregiver burden'/de OR (care* NEAR/3 (burden OR needs)):ti,ab,kw) AND ('caregiver burden'/de OR (burden* OR impact* OR needs OR experience* OR support):ti,ab,kw)

CINAHL via EbscoHost:

#	Query	Resultaten
S1	(MH ("Adolescence+" OR "young adult") OR TI (adolescent* OR "young adult*" OR teen OR teenage* OR AYA# OR TYA# OR "emerging adult*") OR AB (adolescent* OR "young adult*" OR teen OR teenage* OR AYA# OR TYA# OR "emerging adult*"))	764,241
S2	(MH (neoplasms+) OR TI (neoplas* OR tumo#r# OR cancer# OR malignan* OR metasta* OR carcinoma# OR melanoma# OR sarcoma# OR leuk#emia# OR lymphoma#) OR AB (neoplas* OR tumo#r# OR cancer# OR malignan* OR metasta* OR carcinoma# OR melanoma# OR sarcoma# OR leuk#emia# OR lymphoma#))	872,310
S3	(MH ("caregivers+" OR "Caregiver Burden+") OR TI (caregiver# OR "care giver#") OR TI (informal N2 (care* OR support*)) OR TI ((family OR families OR spous* OR partner# OR parent* OR sibling# OR friend# OR relative# OR "loved one#") N2 (carer* OR support*)) OR TI (care* N2 (burden OR needs)) OR AB (caregiver# OR "care giver#") OR AB (informal N2 (care* OR support*)) OR AB ((family OR families OR spous* OR partner# OR parent* OR sibling# OR friend# OR relative# OR "loved one#") N2 (carer* OR support*)) OR AB (care* N2 (burden OR needs)))	141,122
S4	(MH ("Caregiver Burden+") OR TI (burden* OR impact* OR needs OR experience* OR support) OR AB (burden* OR impact* OR needs OR experience* OR support))	1,579,537
S5	S1 AND S2 AND S3 AND S4	1,492

Single line search:

(MH ("Adolescence+" OR "young adult") OR TI (adolescent* OR "young adult*" OR teen OR teenage* OR AYA# OR TYA# OR "emerging adult*") OR AB (adolescent* OR "young adult*" OR teen OR teenage* OR AYA# OR TYA# OR "emerging adult*")) AND (MH (neoplasms+) OR TI (neoplas* OR tumo#r# OR cancer# OR malignan* OR metasta* OR carcinoma# OR melanoma# OR sarcoma# OR leuk#emia# OR lymphoma#) OR AB (neoplas* OR tumo#r# OR cancer# OR malignan* OR metasta* OR carcinoma# OR melanoma# OR sarcoma# OR leuk#emia# OR lymphoma#)) AND (MH ("caregivers+" OR "Caregiver Burden+") OR TI (caregiver# OR "care giver#") OR TI (informal N2 (care* OR support*)) OR TI ((family OR families OR spous* OR partner# OR parent* OR sibling# OR friend# OR relative# OR "loved one#") N2 (carer* OR support*)) OR TI (care* N2 (burden OR needs)) OR AB (caregiver# OR "care giver#") OR AB (informal N2 (care* OR support*)) OR AB ((family OR families OR spous* OR partner# OR parent* OR sibling# OR friend# OR relative# OR "loved one#") N2 (carer* OR support*)) OR AB (care* N2 (burden OR needs))) AND (MH ("Caregiver Burden+") OR TI (burden* OR impact* OR needs OR experience* OR support) OR AB (burden* OR impact* OR needs OR experience* OR support))

Ovid PsycInfo

#	Query	Hits
1	early adolescence/	
2	emerging adulthood/	
3	adolescent*.ti,ab,id.	
4	"young adult*".ti,ab,id.	
5	teen.ti,ab,id.	
6	teenage*.ti,ab,id.	
7	AYA.ti,ab,id.	
8	AYAs.ti,ab,id.	
9	TYA?.ti,ab,id.	
10	"emerging adult*".ti,ab,id.	
11	OR/1-10 [AYAs]	
12	exp neoplasms/	
13	neoplas*.ti,ab,id.	
14	tumo?r?.ti,ab,id.	
15	cancer?.ti,ab,id.	
16	malignan*.ti,ab,id.	
17	metasta*.ti,ab,id.	
18	carcinoma?.ti,ab,id.	
19	melanoma?.ti,ab,id.	
20	sarcoma?.ti,ab,id.	
21	leuk?emia?.ti,ab,id.	
22	lymphoma?.ti,ab,id.	
23	OR/12-22 [cancer]	
24	caregivers/	
25	caregiver?.ti,ab,id.	
26	"care giver?".ti,ab,id.	
27	(informal ADJ3 (care* OR support*)).ti,ab,id.	
28	((family OR families OR spous* OR partner? OR parent* OR sibling? OR friend? OR relative? OR "loved one?") ADJ3 (carer* OR support*)).ti,ab,id.	
29	"caregiver burden"/	
30	(care* ADJ3 (burden OR needs)).ti,ab,id.	
31	OR/24-30 [informal caregivers]	
32	"caregiver burden"/	
33	burden*.ti,ab,id.	

34	impact*.ti,ab,id.	
35	needs.ti,ab,id.	
36	experience*.ti,ab,id.	
37	support.ti,ab,id.	
38	OR/32-37 [burden]	
39	11 AND 23 AND 31 AND 38 [AYAs AND cancer AND informal caregivers AND burden]	

Single line search:
((early adolescence/ OR emerging adulthood/) OR (adolescent* OR "young adult*" OR teen OR teenage* OR AYA OR AYAs OR TYA? OR "emerging adult*").ti,ab,id.) AND (exp neoplasms/ OR (neoplas* OR tumo?r? OR cancer? OR malignan* OR metasta* OR carcinoma? OR melanoma? OR sarcoma? OR leuk?emia? OR lymphoma?).ti,ab,id.) AND ((caregivers/ OR "caregiver burden"/) OR (caregiver? OR "care giver?").ti,ab,id. OR (informal ADJ3 (care* OR support*)).ti,ab,id. OR ((family OR families OR spous* OR partner? OR parent* OR sibling? OR friend? OR relative? OR "loved one?") ADJ3 (carer* OR support*)).ti,ab,id. OR (care* ADJ3 (burden OR needs)).ti,ab,id.) AND ("caregiver burden"/ OR (burden* OR impact* OR needs OR experience* OR support).ti,ab,id.)

2022-10-25:

Ovid MEDLINE(R) ALL <1946 to October 25, 2022>

#	**Query**	**Hits**
1	adolescent/	2192073
2	"young adult"/	999125
3	adolescent*.ti,ab,kf.	310162
4	"young adult*".ti,ab,kf.	115609
5	teen.ti,ab,kf.	6309
6	teenage*.ti,ab,kf.	24036
7	AYA?.ti,ab,kf.	2579
8	TYA?.ti,ab,kf.	611
9	"emerging adult*".ti,ab,kf.	3823
10	or/1-9 [AYA]	2775648
11	exp neoplasms/	3752501
12	neoplas*.ti,ab,kf.	439105
13	tumo?r?.ti,ab,kf.	1914166
14	cancer?.ti,ab,kf.	2139039
15	malignan*.ti,ab,kf.	660929
16	metasta*.ti,ab,kf.	598895
17	carcinoma?.ti,ab,kf.	743517
18	melanoma?.ti,ab,kf.	133911
19	sarcoma?.ti,ab,kf.	104502
20	leuk?emia?.ti,ab,kf.	282012
21	lymphoma?.ti,ab,kf.	198748
22	or/11-21 [cancer]	4986584
23	"caregiver burden"/	489
24	"needs assessment"/	32269
25	(burden* or impact or needs or experience* or support).ti,ab,kf.	3883808
26	or/23-25 [caregiver burden]	3898092
27	10 and 22 and 26	70358
28	(informal adj3 (care* or support*)).ti,ab,kf.	9664
29	(non-medical adj3 (care* or support*)).ti,ab,kf.	335
30	(family adj3 (care* or support*)).ti,ab,kf.	46482

31	(families adj3 (care* or support*)).ti,ab,kf.	12386
32	(spous* adj3 (care* or support*)).ti,ab,kf.	3180
33	(partner? adj3 (care* or support*)).ti,ab,kf.	6209
34	(parent? adj3 (care* or support*)).ti,ab,kf.	17188
35	(sibling? adj3 (care* or support*)).ti,ab,kf.	898
36	(friend? adj3 (care* or support*)).ti,ab,kf.	3500
37	(relative* adj3 (care* or support*)).ti,ab,kf.	8565
38	("loved one?" adj3 (care* or support*)).ti,ab,kf.	589
39	caregiver/ and (informal or non-medical or family or families or spous* or partner? or parent? or sibling? or friend? or relative* or "loved one?").ti,ab,kf.	29352
40	caregiver?.ti,ab,kf.	84446
41	"caregiver burden"/	489
42	(care* adj3 burden).ti,ab,kf.	13397
43	or/28-42 [informal caregiver]	160860
44	27 and 43	1922

Ovid MEDLINE(R) ALL <1946 to October 13, 2022>		
#	**Query**	**Hits**
1	adolescent/	2190326
2	"young adult"/	998313
3	adolescent*.ti,ab,kf.	309096
4	"young adult*".ti,ab,kf.	115217
5	teen.ti,ab,kf.	6290
6	teenage*.ti,ab,kf.	23982
7	AYA?.ti,ab,kf.	2556
8	TYA?.ti,ab,kf.	609
9	"emerging adult*".ti,ab,kf.	3798
10	or/1-9 [AYA]	2773210
11	exp neoplasms/	3745710
12	neoplas*.ti,ab,kf.	438294
13	tumo?r?.ti,ab,kf.	1909325
14	cancer?.ti,ab,kf.	2132044
15	malignan*.ti,ab,kf.	659216
16	metasta*.ti,ab,kf.	597277
17	carcinoma?.ti,ab,kf.	741912
18	melanoma?.ti,ab,kf.	133601
19	sarcoma?.ti,ab,kf.	104311
20	leuk?emia?.ti,ab,kf.	281502
21	lymphoma?.ti,ab,kf.	198329
22	or/11-21 [cancer]	4975288
23	10 and 22 [AYA and cancer]	394126
24	(informal adj3 (care* or support*)).ti,ab,kf. and 23	86
25	(non-medical adj3 (care* or support*)).ti,ab,kf. and 23	3
26	(family adj3 (care* or support*)).ti,ab,kf. and 23	797
27	(families adj3 (care* or support*)).ti,ab,kf. and 23	221
28	(spous* adj3 (care* or support*)).ti,ab,kf. and 23	39
29	(partner? adj3 (care* or support*)).ti,ab,kf. and 23	83
30	(parent? adj3 (care* or support*)).ti,ab,kf. and 23	439

31	(sibling? adj3 (care* or support*)).ti,ab,kf. and 23	68
32	(friend? adj3 (care* or support*)).ti,ab,kf. and 23	69
33	(relative* adj3 (care* or support*)).ti,ab,kf. and 23	119
34	("loved one?" adj3 (care* or support*)).ti,ab,kf. and 23	6
35	informal.ti,ab,kf. and caregiver/ and 23	64
36	non-medical.ti,ab,kf. and caregiver/ and 23	4
37	family.ti,ab,kf. and caregiver/ and 23	421
38	families.ti,ab,kf. and caregiver/ and 23	176
39	spous*.ti,ab,kf. and caregiver/ and 23	44
40	partner?.ti,ab,kf. and caregiver/ and 23	45
41	parent?.ti,ab,kf. and caregiver/ and 23	206
42	sibling?.ti,ab,kf. and caregiver/ and 23	30
43	friend?.ti,ab,kf. and caregiver/ and 23	39
44	relative*.ti,ab,kf. and caregiver/ and 23	93
45	"loved one?".ti,ab,kf. and caregiver/ and 23	15
46	(primary adj3 (carer? or caregiver? or caretaker?)).ti,ab,kf. and 23	116
47	"caregiver burden"/ and 23	8
48	(care* adj3 burden).ti,ab,kf. and 23	186
49	or/24-48	2067
50	"caregiver burden"/ or "needs assessment"/ or (burden* or impact or needs or experience* or support).ti,ab,kf. [burden/needs]	3883331
51	26 and 50	643
52	27 and 50	183
53	30 and 50	334
54	37 and 50	342
55	38 and 50	141
56	41 and 50	157

Embase.com (2022-10-19):

#	Query	Results
1	'adolescent'/exp	1855603
2	'young adult'/exp	477239
3	adolescent*:ti,ab,kw	408942
4	'young adult*':ti,ab,kw	153465
5	teen:ti,ab,kw	8389
6	teenage*:ti,ab,kw	34135
7	aya$:ti,ab,kw	4812
8	tya$:ti,ab,kw	369
9	'emerging adult*':ti,ab,kw	4291
10	#1 OR #2 OR #3 OR #4 OR #5 OR #6 OR #7 OR #8 OR #9	2325863
11	'neoplasm'/exp	5783684
12	neoplas*:ti,ab,kw	530291
13	tumo$r$:ti,ab,kw	2632338
14	cancer$:ti,ab,kw	3063696
15	malignan*:ti,ab,kw	953915
16	metasta*:ti,ab,kw	876983
17	carcinoma$:ti,ab,kw	1014628
18	melanoma$:ti,ab,kw	189764

19	sarcoma$:ti,ab,kw	130878
20	leuk$emia$:ti,ab,kw	395494
21	lymphoma$:ti,ab,kw	294472
22	#11 OR #12 OR #13 OR #14 OR #15 OR #16 OR #17 OR #18 OR #19 OR #20 OR #21	6887368
23	'informal care'/de	59
24	'informal caregiver'/de	46
25	'informal caregiving'/de	17
26	(informal NEAR/3 (care* OR support*)):ti,ab,kw	11910
27	('non medical' NEAR/3 (care* OR support*)):ti,ab,kw	639
28	(family NEAR/3 (care* OR support*)):ti,ab,kw	60554
29	(families NEAR/3 (care* OR support*)):ti,ab,kw	16812
30	(spous* NEAR/3 (care* OR support*)):ti,ab,kw	4095
31	(partner$ NEAR/3 (care* OR support*)):ti,ab,kw	8368
32	(parent$ NEAR/3 (care* OR support*)):ti,ab,kw	22830
33	(sibling$ NEAR/3 (care* OR support*)):ti,ab,kw	1203
34	(friend$ NEAR/3 (care* OR support*)):ti,ab,kw	4448
35	(relative* NEAR/3 (care* OR support*)):ti,ab,kw	11016
36	('loved one$' NEAR/3 (care* OR support*)):ti,ab,kw	955
37	'caregiver'/de AND (informal:ti,ab,kw OR 'non medical':ti,ab,kw OR family:ti,ab,kw OR families:ti,ab,kw OR spous*:ti,ab,kw OR partner$:ti,ab,kw OR parent$:ti,ab,kw OR sibling$:ti,ab,kw OR friend$:ti,ab,kw OR relative*:ti,ab,kw OR 'loved one$':ti,ab,kw)	53844
38	(primary NEAR/3 (carer$ OR caregiver$ OR caretaker$)):ti,ab,kw	8650
39	'caregiver burden'/de	9781
40	(care* NEAR/3 burden):ti,ab,kw	19949
41	#23 OR #24 OR #25 OR #26 OR #27 OR #28 OR #29 OR #30 OR #31 OR #32 OR #33 OR #34 OR #35 OR #36 OR #37 OR #38 OR #39 OR #40	165444
42	#10 AND #22 AND #41	2404
43	#42 NOT ('conference abstract'/it OR 'conference paper'/it OR 'conference review'/it)	1722
44	#42 AND ('conference abstract'/it OR 'conference paper'/it OR 'conference review'/it)	682

CINAHL (19-10-2022)

#	**Query**	**Resultaten**
**S4**	S1 AND S2 AND S3	1,480
**S3**	(MH ("caregivers+" OR "Caregiver Burden+") OR TI ((informal OR non-medical OR family OR families OR spous* OR partner# OR parent# OR sibling# OR friend# OR relative* OR "loved one#") N3 (care* OR support*)) OR TI (primary N3 (carer# OR caregiver# OR caretaker#)) OR TI (care* N3 burden) OR AB ((informal OR non-medical OR family OR families OR spous* OR partner# OR parent# OR sibling# OR friend# OR relative* OR "loved one#") N3 (care* OR support*)) OR AB (primary N3 (carer# OR caregiver# OR caretaker#)) OR AB (care* N3 burden))	118,504
**S2**	(MH (neoplasms+) OR TI (neoplas* OR tumo#r# OR cancer# OR malignan* OR metasta* OR carcinoma# OR melanoma# OR sarcoma# OR leuk#emia# OR lymphoma#) OR AB (neoplas* OR tumo#r# OR cancer# OR malignan* OR metasta* OR carcinoma# OR melanoma# OR sarcoma# OR leuk#emia# OR lymphoma#))	871,017

**S1**	(MH ("young adult" OR "Adolescence+") OR TI (adolescent* OR "young adult*" OR teen OR teenage* OR AYA# OR TYA# OR "emerging adult*") OR AB (adolescent* OR "young adult*" OR teen OR teenage* OR AYA# OR TYA# OR "emerging adult*"))	763,372

Numbers FLOW chart:

Database		Numbers
Medline via Ovid		2190
Embase.com		1787
CINAHL via Ebsco		1471
PsycInfo via Ovid		319
	**Total**	**5767**
	Duplicates	2595
	**Unique references**	**3172**

Conference abstracts:

Database	Numbers
Embase.com	754
